# Complete Coding Sequences of Lumpy Skin Disease Virus Strains Isolated from Cutaneous Lesions in Namibian Cattle during 2016 Outbreaks

**DOI:** 10.1128/MRA.00124-20

**Published:** 2020-07-09

**Authors:** Elisabetta Di Felice, Chiara Pinoni, Siegfried Khaiseb, Cesare Camma, Andrea Capobianco Dondona, Andrea Polci, Umberto Molini, Federica Monaco

**Affiliations:** a Istituto Zooprofilattico Sperimentale dell’Abruzzo e del Molise G. Caporale, Teramo, Italy; b Central Veterinary Laboratory, Windhoek, Namibia; c Department of Pathobiology, School of Veterinary Medicine, University of Namibia, Windhoek, Namibia; DOE Joint Genome Institute

## Abstract

Between September and October 2016, an outbreak of lumpy skin disease (LSD) was monitored in the Okakarara veterinary district of Namibia. The complete coding sequences were obtained for LSD virus isolates from skin nodules from two symptomatic animals.

## ANNOUNCEMENT

Lumpy skin disease (LSD) is a disease of cattle that is transmitted by blood-feeding vectors and is caused by the LSD virus (LSDV), a DNA virus belonging to the genus *Capripoxvirus* (family *Poxviridae*). LSD is endemic in most African countries. Since 2012, the disease has been spreading throughout the Middle East, Greece, and the Balkans (1–3). There are only 14 complete genome sequences of LSDV available in public databases, referred to as vaccine strains or field outbreak strains from Kenya (4), South Africa (5), Europe, and Russia (6–8), with none from Namibia. Here, we report the coding-complete genome sequences of two LSDV strains isolated during a Namibian outbreak reported on two commercial farms 20 km away from Okakarara village, in the Otjozondjupa region of Namibia.

LSDV DNA was identified from two skin nodules with a commercial reverse transcription-PCR assay (Bio-T LSD kit; BioSellal), and the strains were isolated after three passages on primary ovine testicular cells (9). Total DNA was extracted from the supernatant (BioSprint 96 One-For-All Vet kit; Qiagen) and quantified with the Qubit DNA HS assay kit (Thermo Fisher Scientific). One nanogram of DNA was used for library preparation with the Nextera XT library preparation kit and was sequenced on the NextSeq 500 platform (Illumina) using the NextSeq 500/550 midoutput reagent cartridge v2 (300 cycles and standard 150-bp paired-end reads). Quality control and trimming were performed using the NGS QC Toolkit (10). Reads in the quality-trimmed data were mapped to a bovine genome sequence (bosTau8). All reads that did not map to the bovine genome were assembled into a single contig with a *de novo* approach based on the SPAdes v.3.11 assembler (11), followed by genome refinement performed with Pilon v.1.22 software (12). Consensus sequences underwent BLAST searches against the nonredundant/nucleotide (nr/nt) database to identify homologous sequences (13). Totals of 477,478 (Namibia/9/2016) and 296,664 (Namibia/10/2016) paired-end reads were mapped to the Neethling Warmbaths LW strain (GenBank accession number AF409137), with coverages of 34.36× and 18.5×, respectively.

Nucleotide BLAST analysis (NCBI) of the Namibia/9/2016 (150,586 bp; G+C content, 25.87%) and Namibia/10/2016 (150,523 bp; G+C content, 25.32%) strains showed that these genomes shared ≥99% nucleotide identity with field strains from South Africa in 2003 (GenBank accession number AF409137), Serbia in 2016 (KY702007), Greece in 2015 (KY829023), Israel in 2012 (KX894508), Kazakhstan in 2016 (MN642592), and Russia in 2015 (MH893760) and 2017 (MH646674) and 98.82% identity with vaccine strains (KX764643, KX764645, KX764644, AF409138, MG972412, and MK441838). In comparisons with the Neethling Warmbaths LW isolate, the same 12-nucleotide mutations and two 3-nucleotide indels were identified in both Namibian genomes. Moreover, 5 and 27 additional nucleotide modifications were observed in Namibia/9/2016 and Namibia/10/2016, respectively. The effects of the nucleotide changes are shown in Table 1. The two Namibian isolates showed a 15-nucleotide deletion (TAAGTGGAAGCCAAT) in the terminal noncoding part of the inverted terminal repeat region, which has already been described for the LSDV isolates from Greece in 2015, Serbia in 2016, Russia in 2015 and 2017, and Israel in 2012, suggesting possible stability of this deletion in the circulating strains in past years.

**TABLE 1:** Amino acid modifications of Namibia/9/2016 and Namibia/10/2016, compared with the LSDV reference strain Neethling Warmbaths LW

Gene/region	Amino acid change(s)^*a*^ for strain:	
	Namibia/9/2016	Namibia/10/2016
LD002		P100Q, F101Y, Y107X,^*b*^ Y108N, C109X, N112K, I114K
LD003	R220K	R220K
LD005	S44L	S44L
LD009	R129K	
LD013a	D275N	D275N
LD017	H59R	H59R
LD021	L35F	L35F
LD025	S324L	S324L
LD027	S235L	S235L
LD033	A157T	A157T
LD035		K223X, G225V
LD042	R385S	K362T, V384G
LD050		F411I
LD057		C58X
LD063		FF143I, K148N, V149G, I151N
LD083	R176K	R176K
LD087	K107E	K107E
LD092	N49K	E78D, L79I, L145F, T153K
LD096	E29 deletion	E29 deletion
LD098	E634K	
LD115		R185L, I186N, D187X
LD119	Q85 deletion	Q85 deletion
LD133		S335C
LD142		D8E, H11N
LD143	R220H	R220H
LD150	P99T	
LD154	R220K	R220K

a The amino acid changes are indicated using the format XnY, where X indicates the amino acid in the Neethling Warmbaths LW strain (GenBank accession number AF409137), n is the amino acid number of the respective gene, and Y is the amino acid in the Namibian strain.

b X, stop codon.

### Data availability.

These coding-complete genomes have been deposited in GenBank under accession numbers MT007950 and MT007951. Raw data have been submitted to the SRA under BioProject number PRJNA639698.
